# Gold(I)···Lanthanide(III) Bonds in Discrete Heterobimetallic Compounds: A Combined Computational and Topological Study

**DOI:** 10.1021/acs.inorgchem.2c02717

**Published:** 2022-12-07

**Authors:** Daniel Blasco, Dage Sundholm

**Affiliations:** † Department of Chemistry, Faculty of Science, University of Helsinki, P.O. Box 55 (A.I. Virtasen aukio 1), FIN-00014Helsinki, Finland; ‡ Departamento de Química, Centro de Investigación en Síntesis Química (CISQ), Universidad de La Rioja, Madre de Dios 53, 26006Logroño, Spain

## Abstract

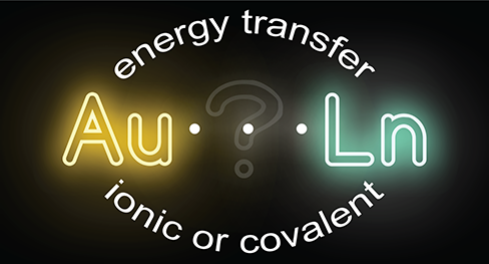

The chemical nature of the ligand-unsupported gold(I)–lanthanide(III) bond in the proposed [Ln^III^(η^5^-Cp)_2_][Au^I^Ph_2_] (**Ln–Au**; Ln^III^ = La^III^, Eu^III^, or Lu^III^; Cp = cyclopentadienide; Ph = phenyl) models is examined from a theoretical viewpoint. The covalent bond-like Au–Ln distances (Au–La, 2.95 Å; Au–Eu, 2.85 Å; Au–Lu, 2.78 Å) result from a strong interaction between the oppositely charged fragments (Δ*E*_int_^MP2^ > 600 kJ mol^–1^), including the aforementioned metal–metal bond and additional Ln^III^–C_ipso_ and C–H···π interactions. The Au–Ln bond has been characterized as a chemical bond rather than a strong metallophilic interaction with the aid of energy decomposition analysis, interaction region indicator, and quantum theory of atoms in molecules topological tools. The chemical nature of the Au–Ln bond cannot be fully ascribed to a covalent or an ionic model; an intermediate situation or a charge shift bond is proposed. The [Au^I^Ph_2_]^−^ anion has also been identified as a suitable lanthanide(III) emission sensitizer for **La–Au** and **Lu–Au**.

## Abstract 2

The nature of the predicted chemical bond between gold(I) and lanthanide(III) and the role of gold(I) fragments as lanthanide(III) emission sensitizers are investigated.

## 1 Introduction

**Scheme 1:**
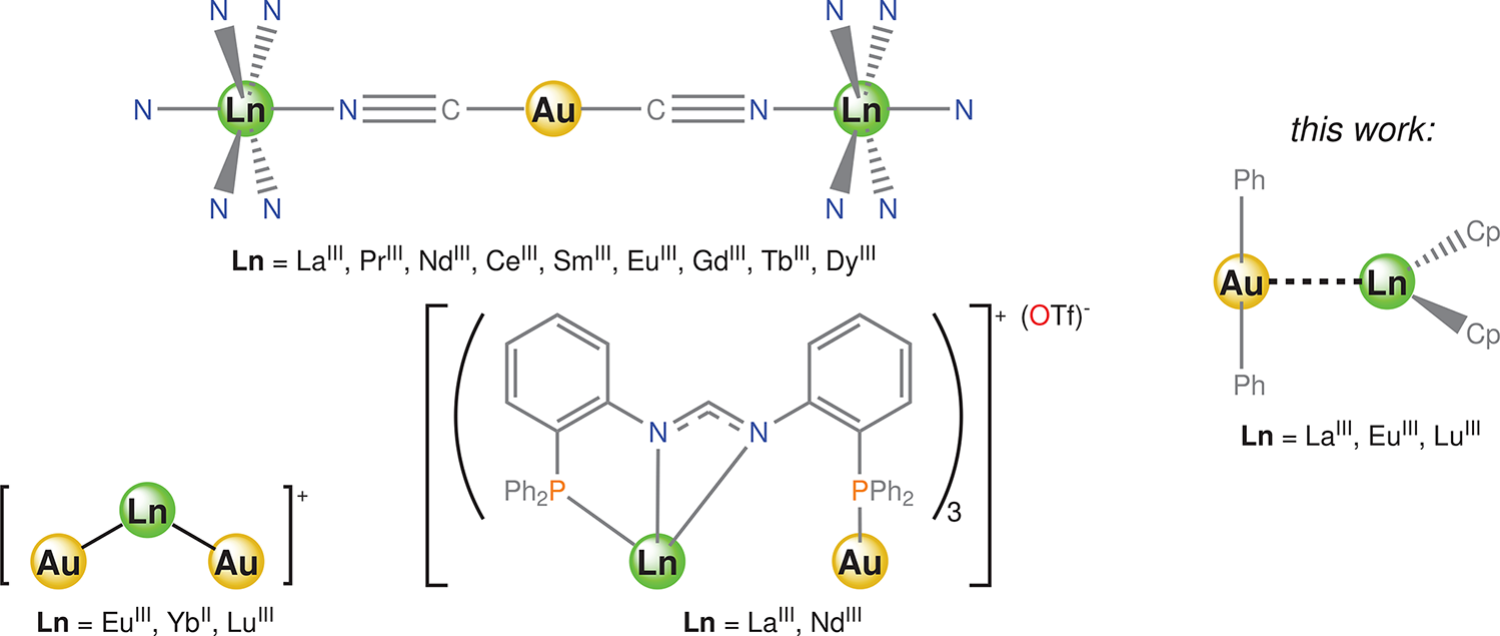
Representative Examples of Heterometallic Gold(I)–Lanthanide(III) Complexes and Theoretical Models Ln^III^[Au^I^(CN)_2_]_3_·3H_2_O (top left, water molecules omitted for the sake of clarity), [Au_2_^–I^Ln^III^]^+^ model (bottom left), [Ln^III^Au^I^(dpfam)_3_]OTf {dpfam = *N,N′*-bis[(2-diphenylphosphino)phenyl]formamidinate; (OTf)^−^ = (CF_3_SO_3_)^−^ (bottom center)}, and the presently studied [Ln^III^(η^5^-Cp)_2_][Au^I^Ph_2_] (**Ln–Au**; Cp = cyclopentadienide; Ph = phenyl; right) models.

The reactivity of the extremely stable dicyanoaurate(I) anion {[Au^I^(CN)_2_]^−^} toward f-block metal(III) salts has recently been a subject of sporadic research that mainly has focused on the efficient sensitization of the monochromatic line-like emission of the lanthanide(III) cations.^1−12^ Other relevant properties such as single-molecule magnetism (SMM)^10^ and birefringence have been examined.^5^ It is accepted, even as textbook knowledge,^13^ that lanthanide-based emission is a result of parity-forbidden radiative 4f–4f transitions [or parity-allowed 4f–5d transitions in the case of, e.g., cerium(III)] because of an energy transfer (ET) from the lowest excited triplet state of the (metallo)ligand, the so-called sensitizer, to the lanthanide atom.^14^ Hence, the global emission efficiency strongly depends on the electronic structure of the sensitizer and the lanthanide, which requires an appropriate energy match for allowing the whole cascade of intersystem crossings (ISCs) and ETs to occur. Several studies have demonstrated that gold(I) and silver(I) dicyanometallates {[M^I^(CN)_2_]^−^; M^I^ = Au^I^ or Ag^I^} and platinum(II) tetracyanometallates {[Pt^II^(CN)_4_]^2–^} are among the most efficient sensitizers because of not only the similarity between their energy levels and those of the lanthanide(III) acceptors but also spin–orbit coupling (SOC) effects. More precisely, the metal···metal-based low-lying triplet states of the aurophilic Au^I^···Au^I^ or metallophilic M^I^(d^10^)···M^II^(d^8^) (M^I^ = Au^I^ or Ag^I^; M^II^ = Pt^II^ or Pd^II^) interactions that are found within Ln^III^[Au^I^(CN)_2_]_3_·3H_2_O [Ln^III^ = La^III^, Pr^III^, Nd^III^, Ce^III^, Sm^III^, Eu^III^, Gd^III^, Tb^III^, or Dy^III^ (Scheme 1, top left)]^7−9,15^ and terbium(III) or europium(III) heterotrimetallic solid state solutions,^2,11^ respectively, have been identified as the best ET donor states. Aurophilic attraction is an extremely important and recurrent structural motif in the chemistry of gold(I)^16−18^ that has been studied theoretically in detail.^19^ It is characterized by unexpectedly short gold(I)···gold(I) distances and high interaction energies of 30–50 kJ mol^–1^. It is relevant not only from a structural point of view but also because it stabilizes reactive intermediates of catalysis.^20^ Its occurrence between gold atoms in higher oxidation states (Au^III^) is still controversial.^21^ The proposals of all of the articles reviewed for the elaboration of this work are based on qualitative interpretations of the luminescence spectra, lifetimes, and quantum yields without any further theoretical support.

Very little is known about a possible direct interaction between gold(I) and lanthanide(III) atoms. In a report by Páez-Hernández et al., they studied the intermetallic bond in the *C*_2*v*_-shaped [Au_2_^–I^Ln^III^]^+^ (Ln^III^ = Eu^III^ or Lu^III^) and [Au_2_^–I^Yb^II^] species (Scheme 1, bottom left) through density functional theory (DFT), complete-active-space self-consistent-field (CASSCF), and second-order complete-active-space perturbation theory (CASPT2) calculations.^22^ They concluded that these Au^–I^–Ln^III^ bonds have a large two-center, two-electron (2c-2e) covalent contribution arising from the Au(6s)–Ln(5d6s) hybridization. Despite being good models for intermetallic compounds,^23,24^ they are poorly descriptive for the purposes of coordination chemistry, where ancillary ligands, especially when dealing with lanthanide cations, play a pivotal role. A recent paper by Roesky et al. illustrated the first example of heterometallic molecular gold(I)–lanthanide(III) complexes without mediation of cyanide ligands.^25^ The authors cleverly employed the tetradentate ligand dpfam {dpfam = *N,N′*-bis[(2-diphenylphosphino)phenyl]formamidinate}, featuring “hard” (nitrogen) and “soft” (phosphorus) donor sites, for a stepwise and selective coordination of lanthanum(III)/neodimium(III) and gold(I) (Scheme 1, bottom center). The intermetallic distances determined by X-ray diffraction are in the limit for the van der Waals interaction (La^III^···Au^I^, 4.24 Å; Nd^III^···Au^I^, 4.20 Å). However, they proposed a possible metal···metal interaction in the first excited state.

To date, an experimental proof of ground state gold(I)–lanthanide(III) bonds and/or interactions is lacking, which is even more striking when considering the plethora of structurally authenticated combinations of other main- and d-block metals with lanthanides and even with actinides.^26^ In a particularly impressive example, a naked lanthanide(III) atom is stabilized by only metal···metal bonds with three rhenocene {[Re^I^(η^5^-Cp)_2_]^−^} anions.^27^ The aforementioned lack of knowledge is probably due to the choice of the coordinating dicyanoaurate(I) anion in almost all studies dealing with this subject. To address it and also to unveil the possible derived optical consequences, we herein present a computational and topological study of three models featuring an unsupported interaction between the noncoordinating diphenylaurate(I) anion ([Au^I^Ph_2_]^−^) and bis(η^5^-cyclopentadienide)lanthanide(III) cations {[Ln^III^(η^5^-Cp)_2_]^+^; Ln^III^ = La^III^, Eu^III^, or Lu^III^} henceforth designated, for the sake of brevity, as **Ln–Au** (Scheme 1, right). With regard to the choice of the [Ln^III^(η^5^-Cp)_2_]^+^ component, closed-shell lanthanum(III) ([Xe]) and lutetium(III) ([Xe] 4f^14^) are adopted to favor possible metallophilic interactions, whereas europium(III) ([Xe] 4f^6^) was considered a suitable representative of open-shell lanthanide cations. Cyclopentadienide ancillary ligands are ideal as each one saturates three coordination vacancies of the highly electron-demanding lanthanide(III) cation while being addressed as spectator ligands.

## 2 Computational Details

All calculations were carried out using TURBOMOLE version 7.5.1.^28,29^ The optimized molecular structures and orbitals were visualized and rendered using the latest version of UCSF ChimeraX.^30^**Ln–Au** models were built from scratch and optimized without any symmetry constraints at the DFT level using the PBE0 functional^31−33^ and the def2-TZVP basis sets.^34,35^ Dispersion interactions were considered with the semiempirical D3(BJ) correction.^36,37^ The resolution-of-the-identity (RI) approximation^38−41^ was used to accelerate the calculations. Effective core potentials (def2-ECP) were used for gold and the lanthanides [Au, 60 core electrons (CE); La, 46 CE; Eu, 28 CE; Lu, 28 CE].^42^ All structures were verified as true local minima by computing the vibrational frequencies.^43^

The gold(I)–lanthanide(III) interaction energies (Δ*E*_int_ in eq 1) were calculated at the restricted Hartree–Fock (RHF/def2-TZVP)^44^ and second-order Møller–Plesset perturbation theory (MP2/def2-TZVP)^45−47^ levels using eq 1 for the counterpoise correction to the basis set superposition error (BSSE).^48,49^
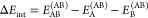
1where *E*_AB_^(AB)^, *E*_A_^(AB)^, and *E*_B_^(AB)^ are the energies of the complex and the fragments calculated using the basis sets of the complex.

According to energy decomposition analysis (EDA),^50^ the instantaneous interaction energy between two fragments (Δ*E*_int_ in eq 2) can be partitioned into the sum of quasiclassical electrostatic , exchange–repulsion , orbital relaxation , correlation , and dispersion  contributions:
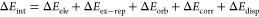
2

Whereas the physical meaning of the Δ*E*_ele_, Δ*E*_corr_, and Δ*E*_disp_ contributions is self-explanatory, Δ*E*_ex–rep_ (or Pauli repulsion, Δ*E*_Pauli_) accounts for the destabilizing interaction between electrons of the same spin and Δ*E*_orb_ stands for orbital relaxation and mixing between fragments. The present formulation of EDA is implemented in TURBOMOLE only at the RHF and DFT levels of theory.

The quantum theory of atoms in molecules (QTAIM)^51,52^ electron-density descriptors ρ_e_(**r**), , and *H*, and the recently proposed interaction region indicator (IRI, eq 3),^53^ have been computed with the latest version of Multiwfn.^54^ The IRI isosurfaces
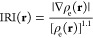
3have been plotted with VMD.^55^

The optimized molecular structures of the ground state were used in time-dependent DFT (TD-DFT)^56−60^ calculations of the first vertical singlet (S_1_ ← S_0_) and triplet (T_1_ ← S_0_) excitation energies using the PBE0 functional^31−33^ and the def2-TZVP basis sets.^34,35^

## 3 Results and Discussion

### 3.1 Structure Optimization

**Figure 1:**
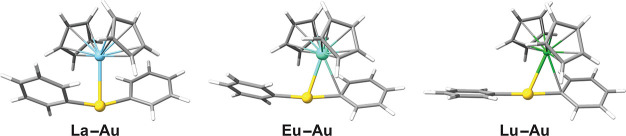
Molecular structures of the **Ln–Au** models. Color code: C, gray; H, white; Au, yellow; Eu, aquamarine blue; La, sky blue; Lu, green.

The molecular structure of the **Ln–Au** models optimized at the PBE0/D3(BJ)/def2-TZVP level is shown in Figure 1. A selection of bond lengths and bond angles are listed in Table 1. We obtained remarkably short Au^I^···Ln^III^ distances in contrast to recurrent measurements of nonbonding distances between the metals, which may be a consequence of the choice of the ancillary ligands attached to gold(I) (phenyl vs cyanide) and lanthanide(III) (cyclopentadienide vs aquo, nitrate, and/or polypyridines). The overall stability of the rigid framework, which is driven by, e.g., the intermetallic μ^2^-cyanido coordination, probably overrules the stability of single Au^I^···Ln^III^ bonds and/or interactions.

**Table 1:** Selected Bond Lengths (in angstroms) and Angles (in degrees) of the **Ln–Au** Models, with *R*_Au–Ln_ Distances (in angstroms) According to the Additive Covalent Radius Convention^a^,^b^

	Au^I^···Ln^III^	Au^I^–C_ipso_	Ln^III^–C_ipso_	Ln^III^–C_meta_	C_ipso_–Au^I^–C_ipso_
**La–Au**	2.95 (3.04),^a^ (3.43)^b^	2.06	3.28	–	159
**Eu–Au**	2.85 (2.92),^a^ (3.34)^b^	2.04, 2.09	2.83, 3.90	2.84	172
**Lu–Au**	2.78 (2.86),^a^ (3.23)^b^	2.05, 2.11	2.69, 4.01	2.75	176

a Data from refs (61) and (62).

b Data from ref (63).

When the computed Au^I^···Ln^III^ distances are compared to the sum of the single-bond covalent radii (eq 4) of the involved atoms
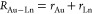
4one obtains a coarse idea of the covalent character of the Au–Ln bond. Table 1 also includes *R*_Au–Ln_ values calculated from the data sets of covalent radii of Pyykkö et al.^61,62^ and Cordero et al.^63^ The decreasing trend in the Au^I^···Ln^III^ distances with an increase in the atomic number of Ln^III^ [*d*(Au–La) > *d*(Au–Eu) > *d*(Au–Lu)] agrees with the lanthanide contraction effect.^13^ The computed Au^I^···Ln^III^ distances are roughly 0.1 Å shorter than the Au–Ln estimates. Thus, a single covalent bond is more likely than a metallophilic interaction between the gold(I) and lanthanide(III) atoms. The Wiberg bond indices (WBIs) of the Au–Ln bond and other WBIs of interest are listed in Table 2. The WBI values are >0.6, suggesting that there is a covalent bond between the metals. The WBI of the Au–Ln bond of the **Ln–Au** models can be compared to those reported by Roesky et al. for the [La^III^Au^I^(dpfam)_3_]^+^ cation.^25^ Their WBI values calculated at different DFT levels of theory range between 0.12 [BP/D3(BJ)/def2-SVP] and <0.02 [PBE0/D3(BJ)/def2-TZVP*]. These values are small in comparison to the WBI value of the **Ln–Au** models.

**Table 2:** Selected WBIs (PBE0/D3(BJ)/def2-TZVP level of theory) of the **Ln–Au** Models

	Au^I^···Ln^III^	Au^I^–C_ipso_	Ln^III^–C_ipso_	Ln^III^–C_meta_
La–Au	0.62	0.67, 0.67	<0.10	–
Eu–Au	0.63	0.48, 0.75	0.19	0.19
Lu–Au	0.68	0.43, 0.72	0.19	0.18

The effective charges of the gold(I) and lanthanide(III) atoms in the **Ln–Au** models have been calculated using the natural population analysis (NPA) approach. The charges are compared in Table 3 to those of the corresponding isolated [Ln^III^(η^5^-Cp)_2_]^+^ (model **Ln**) and [Au^I^Ph_2_]^−^ (model **Au**) ions optimized at the same level of theory [PBE0/D3(BJ)/def2-TZVP]. In the isolated ions as well as in the **Ln–Au** models, the metals have an effective charge that is much smaller than their formal oxidation state. In the formation of the Au–Ln bond, electron density is transferred from gold(I) to lanthanide(III). The NPA charge of gold(I) increases by ∼0.2. The positive charge of the lanthanide(III) atom in **Eu–Au** and **Lu–Au** decreases more than it increases in gold(I), suggesting a further stabilizing role of the Ln^III^–C_ipso_ bonds.

**Table 3:** NPA Charges (PBE0/D3(BJ)/def2-TZVP level of theory) of the **Ln**, **Au**, and **Ln–Au** Models

	Ln^III^	Au^I^	Δ(Ln^III^)^a^	Δ(Au^I^)^a^
**La**	1.93	–	–	–
**Eu**	1.77	–	–	–
**Lu**	2.03	–	–	–
**Au**	–	0.30	–	–
La–Au	1.75	0.48	–0.18	+0.18
Eu–Au	1.33	0.49	–0.45	+0.18
Lu–Au	1.52	0.49	–0.57	+0.19

a Δ(Ln^III^)(Δ(Au^I^)) = **Ln–Au** – **Ln** (**Au**).

A short metal···metal distance, even if it is not supported by ancillary ligands (as in the present case), is not enough to guarantee that there is a covalent bond. Pyykkö et al. clearly stated that one cannot estimate bond lengths and bonding character for strongly ionic systems by adding the covalent radii.^61,62^ There are some concerns about the pure covalent character of the Au–Ln bond. We note that the f-block ions are “noble gas-like” regarding covalent bonding due to the efficient shielding of the 4f valence orbitals by the outer electron shells.^13^ Because gold is the most electronegative metal of the periodic table (χ_P_ = 2.54),^64^ the combination of the [Au^I^Ph_2_]^−^ anion with highly charged lanthanide(III) cations leads to a significant Coulombic attraction. We therefore carried out an energy decomposition analysis of the Au–Ln bond to elucidate its bonding character (*vide infra*).

There is an evident shift of the [Ln^III^(η^5^-Cp)_2_]^+^ cation with respect to the [Au^I^Ph_2_]^−^ anion from an almost eclipsed conformation in **La–Au** toward formation of a Au^I^–C_ipso_ bond in **Eu–Au** and **Lu–Au**. Depletion of the electron density of C_ipso_ is reflected in a weakening of the Au^I^–C_ipso_ bond, which is suggested by its elongation (Table 1) and the decreasing WBI (Table 2). The presence of the [Ln^III^(η^5^-Cp)_2_]^+^ fragment leads to a significant distortion of the ideal linear environment of the dicoordinated [Au^I^Ph_2_]^−^ anion (Figure 1 and Table 1). In the extreme case, both Au^I^–C_ipso_ bonds would be bent toward the lanthanum(III) atom, suggesting that lanthanide(III) completes its coordination sphere by withdrawing electron density from the Au^I^–C_ipso_ bond or even from C_meta_ in **Eu–Au** and **Lu–Au**. This will be further assessed with the aid of topological tools (*vide infra*).

### 3.2 Energy Decomposition Analysis (EDA)

The counterpoise-corrected interaction energies between the gold(I) and lanthanide(III) fragments calculated at the RHF and MP2 levels are listed in Table 4. We considered the experimentally plausible heterolytic fragmentation into [Au^I^Ph_2_]^−^ and [Ln^III^(η^5^-Cp)_2_]^+^ ions instead of the homolytic dissociation into [Au^I^Ph_2_]• and [Ln^III^(η^5^-Cp)_2_]• radicals.

**Table 4:** Counterpoise-Corrected Interaction Energies of **Ln–Au** (in kilojoules per mole) Calculated at the RHF/def2-TZVP and MP2/def2-TZVP Levels

	Δ*E*_int_^RHF^	Δ*E*_int_^MP2^	Δ*E*_int_^MP2^ – Δ*E*_int_^RHF^ (%)^a^
La–Au	–467.69	–602.99	–135.30 (22.44)
Eu–Au	–456.71	–604.93	–148.23 (24.50)
Lu–Au	–468.49	–630.17	–161.68 (25.66)

a Percentages are calculated with respect to Δ*E*_int_^MP2^.

The interaction energies calculated at the MP2 level are >600 kJ mol^–1^ and are similar for the three molecules. The large interaction energies suggest that there is a bond between the metals and that the short Au–Ln distance is not due to metallophilic interactions between closed-shell gold(I) and lanthanide(III). The binding energy can be compared to typical aurophilic interaction energies of 30–50 kJ mol^–1^, which is of the same the size as the binding energy of strong hydrogen bonds. Aurophilicity is even the strongest metallophilic interaction.^16−18^ The bond between the fragments cannot be assigned to a single interaction. The role of C–H···π interactions cannot be ignored (*vide infra*). The difference between the interaction energies calculated at the RHF  and MP2  levels is the electron correlation contribution to the stabilization of the Au–Ln bond. Electron correlation accounts for >20% of the binding energy at the MP2 level. Because a previous study showed that the wave function of complexes with TM–Ln (TM = transition metal) bonds does not have a significant multiconfiguration character,^65^ we assume that the energy difference can be ascribed to the dispersion interaction between [Au^I^Ph_2_]^−^ and [Ln^III^(η^5^-Cp)_2_]^+^ and dynamical correlation effects on the Au–Ln bond.

EDA splits interaction energies  into contributions from electrostatic interaction , Pauli repulsion , and orbital relaxation . EDA of the interaction energy between [Au^I^Ph_2_]^−^ and [Ln^III^(η^5^-Cp)_2_]^+^ was calculated at the PBE0 level (Table 5). The EDA contributions belong to the chemical bonding vocabulary of today and introduce an analogy with the heuristic ionic and covalent models used among synthetic chemists.^66^ We discuss trends among the **Ln–Au** models rather than providing an absolute EDA partitioning, because there is no obvious relation between the EDA values and periodic trends among the lanthanide ions, which is in contrast to the geometrical parameters listed in Table 1. The electrostatic component is ∼60% of the interaction energy, whereas the orbital relaxation contribution is <30%. We cannot assign the Au–Ln bonds as pure covalent or ionic bonds, which has also been pointed out in previous studies of TM–Ln systems.^27,65,67,68^ The covalent orbital contribution to the bond is significant. However, the contribution from Pauli repulsion is larger with an opposite sign. An appropriate description seems to be that an ionic-reinforced weak covalent bond keeps the fragments together. One should though realize that this an oversimplification of the predicted and presumably also of the experimental bonding character.

**Table 5:** Energy Decomposition Analysis (EDA in kilojoules per mole) of the **Ln–Au** Models Calculated at the PBE0/D3(BJ)/def2-TZVP Level of Theory

	Δ*E*_int_	Δ*E*_ele_ (%)^a^	Δ*E*_ex–rep_	Δ*E*_orb_ (%)^a^	Δ*E*_corr_ (%)^a^	Δ*E*_disp_ (%)^a^
La–Au	–580.42	–468.30 (55.50)	263.31	–240.06 (28.45)	–84.57 (10.02)	–50.79 (6.02)
Eu–Au	–574.50	–525.99 (58.23)	328.87	–249.68 (27.64)	–80.99 (8.97)	–46.71 (5.17)
Lu–Au	–598.53	–550.05 (57.02)	366.08	–276.41 (28.66)	–91.37 (9.47)	–46.78 (4.85)

a The percentages are calculated with respect to the sum of the stabilizing contributions, i.e., Δ*E*_ele_ + Δ*E*_orb_ + Δ*E*_corr_ + Δ*E*_disp_.

### 3.3 Interaction Region Indicator Topological Analysis

The interaction region indicator (IRI) method^53^ is a recently proposed real-space function based on the reduced density gradient (RDG) aiming to visualize covalent and noncovalent interactions. The IRI developers claim that it solves certain flaws of the well-established density overlap regions indicator (DORI).^69^ Because the DORI and IRI isosurfaces are weighted by the sign of the second largest eigenvalue of the Hessian of the electron density [sign(λ_2_)·ρ_e_(**r**)], one can visually distinguish attractive [blue to green, sign(λ_2_)·ρ_e_(**r**) < 0], van der Waals [green, sign(λ_2_)·ρ_e_(**r**) ≈ 0], and repulsive [green to red, sign(λ_2_)·ρ_e_(**r**) > 0] interactions. Thus, IRI is well suited for elucidating the van der Waals or covalent/ionic nature of the Au–Ln bond.

**Figure 2:**
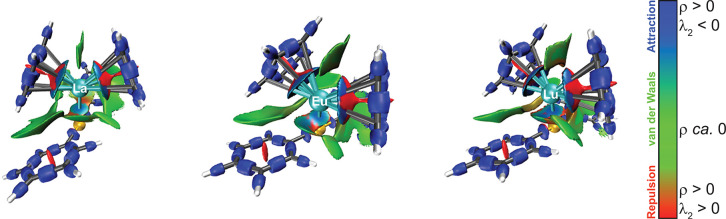
IRI isosurfaces (isovalue = 1.1) considered with  of the **La–Au** (left), **Eu–Au** (center), and **Lu–Au** (right) models. Color code of the atoms: C, gray; H, white; Au, yellow; Ln, cyan. Color code of the IRI isosurfaces: blue, attraction of covalent, hydrogen, halogen, etc., bonds; green, van der Waals interaction; red, repulsion such as steric effects, etc.

**Figure 3:**
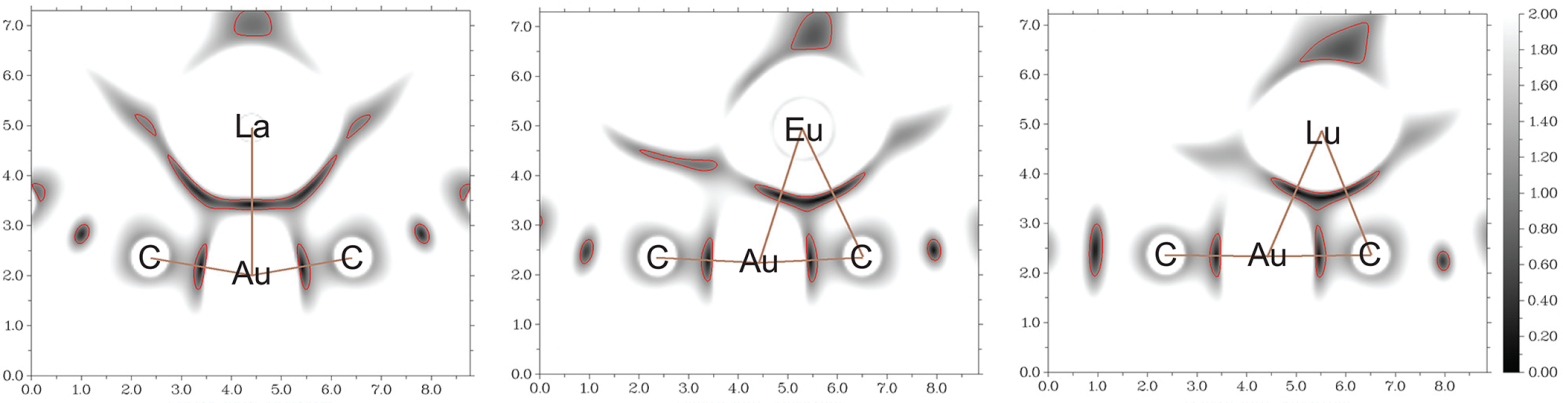
IRI plots of the **La–Au** (left), **Eu–Au** (center), and **Lu–Au** (right) models. Brown lines indicate bonds. Red contours correspond to an isovalue of 1.1. Distances are given in angstroms.

The sign(λ_2_)·ρ_e_(**r**)-mapped IRI isosurfaces of the **Ln–Au** models are shown in Figure 2, whereas Figure 3 depicts the raw IRI functions in the plane containing the C_ipso_–Au^I^–C_ipso_ moiety and the lanthanide(III) atom. The blueish spot in the slab between the metals in Figure 2 corresponds to an electron-rich area  with attractive bonding (λ_2_ < 0). Its position coincides with the intermetallic axis, suggesting that it is chemical bond rather than a van der Waals (metallophilic) interaction. The Ln^III^–C_ipso_ and Ln^III^–C_meta_ bonds are also seen in Figure 2, but a clearer view of them is shown in Figure 3. IRI values close to zero are colored black and indicate a strong interaction, although one cannot discern whether it is attractive or repulsive. The green slabs in Figure 2 show C–H···π interactions between the cyclopentadienide ligands of the lanthanide(III) moiety and the phenyl ligands of the gold(I) moiety as well as the π-stacking interactions between the two cyclopentadienide groups.

The electron density , its Laplacian , and the energy density (*H*) in the bond critical point (BCP) between gold(I) and lanthanide(III) were computed using the QTAIM approach.^51^ The BCP descriptors can be related to the bond type and its strength.^52^ The QTAIM calculations show that there is a BCP along the Au–Ln bond where the gradient norm of the electron density is zero  and two eigenvalues of the Hessian of the electron density are negative. Large ρ_e_(**r**) values (>0.2 au), a negative  indicating accumulated electron density, and a negative *H* represent covalent bonds, whereas small ρ_e_(**r**) values (<0.1 au), a positive  indicating reduced electron density, and a positive *H* indicate that the bond is ionic.^70−72^ The  and *H* values listed in Table 6 have opposite signs, suggesting an intermediate between an ionic and a covalent bond, which agrees with previous results. Thus, according to the QTAIM calculations, the Au–Ln bond is neither ionic nor covalent.

**Table 6:** QTAIM Properties (in atomic units), i.e., Electron Density , Its Laplacian , and Energy Density (*H*), in the BCP of the Au–Ln Bond of the **Ln–Au** Models

	ρ_e_(**r**) × 10	∇_2_[ρ_e_(**r**)]	*H* × 10^2^
La–Au	0.247	0.066	–0.190
Eu–Au	0.258	0.070	–0.214
Lu–Au	0.266	0.072	–0.232

### 3.4 Optical Properties

The electron transfer from the sensitizer to the lanthanide ion is necessary to activate the 4f–4f radiative de-excitation channel of the latter, which is otherwise a forbidden transition due to the Laporte selection rule.^13,14,73^ Even though the energy match between the sensitizer and the lanthanide is the most important factor affecting the quantum yield, the sensitizer–lanthanide distance, spectral overlap, etc., play an important role in the luminescence intensity. In the experimental study by Latva et al., they concluded that the quantum yield is largest when the energy is transferred from the lowest triplet state of the sensitizer to the lowest excited state of lanthanide(III).^74^ The excitation energies of the first excited singlet and triplet states of the **Ln–Au** models were therefore studied at the TD-DFT/PBE0/def2-TZVP level of theory. The discussion is limited to TD-DFT calculations on the **La–Au** and **Lu–Au** models because for the **Eu–Au** model the TD-DFT calculations yielded physically meaningless results, suggesting that TD-DFT/PBE0 calculations are not well-suited for studies of excited states of the **Eu–Au** model.

**Figure 4:**
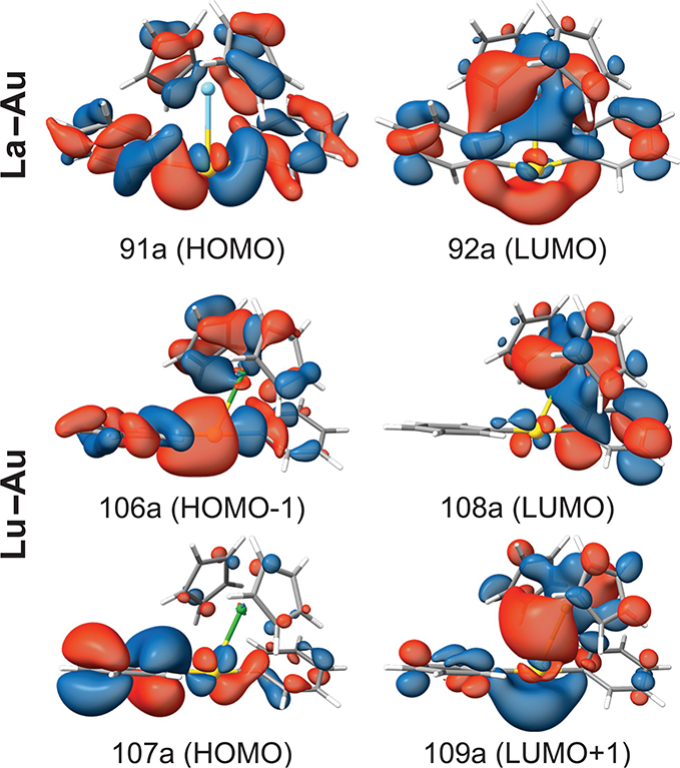
Most relevant molecular orbitals of the **Ln–Au** models (**Ln** = La^III^ or Lu^III^). Color code: C, gray; H, white; Au, yellow; La, sky blue; Lu, green.

The first singlet-to-singlet and singlet-to-triplet vertical excitation energies of the **La–Au** and **Lu–Au** models are listed in Table 7. The molecular orbitals with the largest contribution to these excitations are depicted in Figure 4. The excitation character of the singlet excitation of **La–Au** is completely dominated (97.2%) by the HOMO (91a) to LUMO (92a) transition corresponding to a mixture of intraligand transfers and a charge transfer from [Au^I^Ph_2_]^−^ and Cp^–^ (metallo)ligands to the Au–La bonding region. The contribution of 35% to the excitation character is also the dominant contribution to triplet excitation. The significant population of the LUMO orbital in the lowest singlet and triplet states suggests that there is an effective sensitization of the lanthanum(III) ion. The two main contributions to the singlet state of the **Lu–Au** models are HOMO–1 (106a) to LUMO (108a) and HOMO (107a) to LUMO (108a) with similar weights. The spatial location of the LUMO orbital between the metals is analogous to that of **La–Au**. However, a large number of orbital excitations that are not listed in Table 7 contribute to the T_1_ ← S_0_ transition because the largest contribution is the HOMO (107a) to LUMO+1 (109a) transition that accounts for only 20.5% of the excitation character.

**Table 7:** First Excitation Energies in electronvolts (inverse centimeters) of the **Ln–Au** (**Ln** = La^III^ or Lu^III^) Models Calculated at the TD-DFT/PBE0/def2-TZVP Level^a^

transition	energy	*f*^b^ × 10	contributions (%)	
La–Au				
S_1_ ← S_0_	3.822 (30830)	0.425	91a → 92a	97.2
T_1_ ← S_0_	3.457 (27880)	–	91a → 92a	35.0
Lu–Au				
S_1_ ← S_0_	4.109 (33140)	0.113	106a → 108a	48.8
			107a → 108a	42.3
T_1_ ← S_0_	3.452 (27840)	–	107a → 109a	20.5

a The oscillator strength (*f*) and the dominating excitation character of the excited states are also reported.

b *f* is a mixed representation of the oscillator length and velocity.

## 4 Conclusions

Our calculations demonstrate that gold(I) and lanthanide(III) atoms can chemically bind when suitable noncoordinating ancillary ligands are chosen. This finding opens the field to a whole new family of heterometallic compounds featuring Au–Ln bonds, which may display excellent photophysical quantum yields and intriguing properties such as bimetallic catalysis and/or single-molecule magnetism. We suggest that the Au–Ln bonds belong to the recently proposed class of charge shift (CS) bonds.^75,76^ The CS bonding occurs when electron lone pairs or filled semicore subshells exert a strong Pauli repulsion, weakening and even overruling the covalent contribution to the bond as in, e.g., diatomic fluorine. The resonance energy of the mixing of the pure covalent and ionic nature of the wave function acts as the binding force. A feature that characterizes CS bonds is the combination of significant ρ_e_(**r**) and small positive  values in the BCP. A valence bond (VB) analysis should be performed to assess whether the Au–Ln bonds are of CS type. However, we consider that the Au–Ln bonds belong to this category on the basis of the computed QTAIM properties and the fact that the filled 4d^10^ subshell of the lanthanides may exert a strong Pauli repulsion on the valence 4f orbitals. The proposed nature of the Au–Ln CS bond seems to represent TM–Ln bonding, because analogous intermediate covalent–ionic bonds have been previously described by Butovskii et al.,^27,67,68^ who identified electrostatic attraction as the driving force of Re–Ln bonds, whereas the basin analysis of the electron localizability indicator (ELI-D) suggested a covalent bond between the metals. Further efforts to synthesize and experimentally characterize these complexes will be attempted and will be reported in due time.

## Supporting Information Available

Cartesian coordinates of the **Ln–Au**, **Ln**, and **Au** models (PDF)
