# Supplementary material for: Gold(I)···Lanthanide(III) Bonds in Discrete Heterobimetallic Compounds: A Combined Computational and Topological Study
Source: Inorg Chem. 2022 Dec 7;61(50):20308–15. doi: 10.1021/acs.inorgchem.2c02717 (PMC9768751; doi:10.1021/acs.inorgchem.2c02717)
Supplement: Supplementary file 1 — ic2c02717_si_001.pdf [file ic2c02717_si_001.pdf]

Gold(I)···Lanthanide(III) Bonds in Discrete Heterobimetallic Compounds: A Combined Computational and Topological Study

Daniel Blasco[*a,b] and Dage Sundholm[*a]

[a] Department of Chemistry, Faculty of Science, University of Helsinki, P.O. Box 55 (A.I. Virtasen aukio 1), FIN-00014, Helsinki, Finland

[b] Departamento de Química, Centro de Investigación en Síntesis Química (CISQ), Universidad de La Rioja, Madre de Dios 53, 26006, Logroño, Spain

Supporting Information

1 Cartesian coordinates of model Au.

Au	-0.2373058	0.0647768	-1.0468430
C	-2.2907299	0.1014513	-1.1776679
C	-2.9806120	-0.2530378	-2.3496572
C	-3.1100211	0.4835106	-0.1015547
C	-4.3670441	-0.2290529	-2.4472262
H	-2.4109727	-0.5612459	-3.2224732
C	-4.4973862	0.5131829	-0.1815884
H	-2.6446646	0.7708304	0.8377308
C	-5.1389312	0.1563097	-1.3598059
H	-4.8491657	-0.5134351	-3.3793088
H	-5.0835370	0.8176720	0.6820653
H	-6.2223342	0.1780793	-1.4291287
C	1.8162284	0.0275585	-0.9160386
C	2.5007133	-0.7919034	-0.0020407
C	2.6409550	0.8184048	-1.7343585
C	3.8871289	-0.8256190	0.0911926
H	1.9265369	-1.4310457	0.6636921
C	4.0284553	0.7974538	-1.6544961
H	2.1795392	1.4780838	-2.4645386
C	4.6646718	-0.0280476	-0.7373982
H	4.3657684	-1.4785065	0.8169528
H	4.6185429	1.4311312	-2.3121419
H	5.7481426	-0.0506951	-0.6704291

2 Cartesian coordinates of model La.

C	0.3345625	1.4595504	2.0299106
H	0.2737472	0.4484168	2.4099415
C	1.4889844	2.0845418	1.5170867
C	-0.7101503	2.4119950	2.0314312
H	2.4692827	1.6343128	1.4285303
C	1.1595680	3.4197067	1.1915803
H	-1.7118671	2.2558939	2.4104137
C	-0.2010140	3.6231709	1.5178384
H	1.8437287	4.1724618	0.8250473
H	-0.7404007	4.5558403	1.4358217
C	-0.8283699	2.5970547	-3.2810312
H	-0.8005971	1.8243608	-4.0377757
C	-1.9607147	2.9901027	-2.5392248
C	0.2441103	3.4424340	-2.9163265
H	-2.9533186	2.5668223	-2.6233763
C	-1.5897564	4.0710738	-1.7083764
H	1.2384946	3.4275074	-3.3434073
C	-0.2253506	4.3546056	-1.9480177
H	-2.2486644	4.6283513	-1.0570607
H	0.3421807	5.1607135	-1.5055421
La	-0.2320769	1.9833681	-0.6382444

3 Cartesian coordinates of model Eu.

C	0.4849917	1.4452156	1.8105853
H	0.7297754	0.4163884	2.0417262
C	1.4000315	2.4536502	1.4358139
C	-0.7982773	2.0291148	1.8792472
H	2.4700367	2.3307709	1.3266008
C	0.6833761	3.6583081	1.2722655
H	-1.7105267	1.5249069	2.1714856
C	-0.6776704	3.3954179	1.5461336
H	1.1071121	4.6206394	1.0244339
H	-1.4773815	4.1215348	1.5504644
C	-1.0162680	2.5389553	-3.0643632
H	-1.3238075	1.7031913	-3.6796320
C	-1.8494831	3.2882028	-2.2025929
C	0.2743449	3.1056155	-3.0196361
H	-2.9070939	3.1249198	-2.0408614
C	-1.0743405	4.3176298	-1.6295656
H	1.1325691	2.7771767	-3.5920216
C	0.2419021	4.2006427	-2.1281238
H	-1.4317042	5.0834942	-0.9576848
H	1.0671424	4.8622242	-1.9043555
Eu	-0.1323505	2.1142852	-0.6407007

4 Cartesian coordinates of model Lu.

Lu	0.4977405	1.0790731	0.0876378
C	0.7699160	0.1945704	2.4214147
H	0.6427530	-0.8562702	2.6463084
C	1.9888457	0.8322576	2.0919279
C	-0.2395868	1.1864601	2.4784584
H	2.9557781	0.3523840	2.0146576
C	1.7324947	2.2155438	1.9393832
H	-1.2757095	1.0252191	2.7444521
C	0.3556642	2.4335517	2.1827988
H	2.4669297	2.9790160	1.7232513
H	-0.1450420	3.3906898	2.1769311
C	-0.0715577	1.1824493	-2.3562899
H	0.0467830	0.3241293	-3.0044955
C	-1.2482360	1.5398762	-1.6552050
C	0.8950758	2.1911446	-2.1255577
H	-2.1827951	0.9943104	-1.6634451
C	-1.0058291	2.7600278	-0.9844431
H	1.8871076	2.2309344	-2.5560321
C	0.3203344	3.1631802	-1.2761200
H	-1.7204769	3.3112212	-0.3901494
H	0.7941383	4.0744944	-0.9403462

5 Cartesian coordinates of model La–Au.

Au	-0.2413798	-0.8956271	-1.2582056
C	-2.2639224	-0.4924578	-1.2813056
C	-3.0820653	-0.5729786	-2.4172262
C	-2.8098644	0.1938900	-0.1797727
C	-4.3508914	-0.0124756	-2.4519003
H	-2.7172437	-1.0834562	-3.3025142
C	-4.0737565	0.7799474	-0.2117399
H	-2.2554836	0.2327147	0.7592231
C	-4.8499477	0.6786954	-1.3538896
H	-4.9545096	-0.1026112	-3.3491773
H	-4.4534092	1.2991668	0.6623057
H	-5.8358927	1.1274183	-1.3902211
C	1.7846672	-0.5585094	-1.0730463
C	2.5989216	-1.1124314	-0.0750288
C	2.3391490	0.5200368	-1.7882235
C	3.8722113	-0.6282316	0.1891818
H	2.2271581	-1.9421176	0.5173021
C	3.6073981	1.0290360	-1.5156718
H	1.7880637	0.9513677	-2.6256258
C	4.3795709	0.4539256	-0.5205530
H	4.4727002	-1.0898644	0.9662662
H	3.9933786	1.8623773	-2.0939000
H	5.3691262	0.8380409	-0.3013390
C	0.3360640	1.4472441	2.0561443
H	0.2610900	0.4135061	2.3633583
C	1.4932764	2.0741087	1.5560704
C	-0.6992141	2.4068628	2.0906894
H	2.4513248	1.5983476	1.3953956
C	1.1754529	3.4205600	1.2784036
H	-1.7058974	2.2445218	2.4515767
C	-0.1809209	3.6272942	1.6114808
H	1.8587739	4.1703921	0.9041363
H	-0.7181962	4.5622435	1.5381416
C	-0.8318911	2.5961931	-3.3100625
H	-0.7914586	1.7729996	-4.0094864
C	-1.9647228	2.9952985	-2.5749802
C	0.2310816	3.4631729	-2.9763423
H	-2.9347892	2.5174284	-2.6001118
C	-1.6040754	4.1067911	-1.7843727
H	1.2289000	3.4378692	-3.3930396
C	-0.2450926	4.3976065	-2.0337126
H	-2.2610628	4.6573107	-1.1251483
H	0.3220550	5.2100107	-1.6015709
La	-0.2283190	1.9925230	-0.6373514

6 Cartesian coordinates of model Eu–Au.

Au	0.2289571	-1.9091740	-0.4494123
C	-1.8388983	-1.6235973	-0.5045132
C	-2.5805954	-1.8171606	-1.6797494
C	-2.5633998	-1.1377174	0.5993008
C	-3.9375697	-1.5431946	-1.7497950
H	-2.0807152	-2.1905118	-2.5676236
C	-3.9220428	-0.8302640	0.5341374
H	-2.0691080	-1.0415233	1.5703818
C	-4.6133652	-1.0321875	-0.6472864
H	-4.4737310	-1.7130726	-2.6776027
H	-4.4373577	-0.4563328	1.4124483
H	-5.6704909	-0.8022965	-0.7109175
C	2.2647198	-1.9399491	-0.2833995
C	2.8914844	-2.6010804	0.7806011
C	3.0846550	-1.1510732	-1.1009058
C	4.2519119	-2.4721970	1.0258613
H	2.3037070	-3.2254031	1.4469096
C	4.4448530	-1.0080127	-0.8581738
H	2.6524242	-0.6235479	-1.9464118
C	5.0338578	-1.6645214	0.2123496
H	4.7024285	-2.9968014	1.8620835
H	5.0469126	-0.3784859	-1.5052557
H	6.0935481	-1.5496349	0.4106013
C	0.5159038	0.1539921	2.5930603
H	0.4789964	-0.8843403	2.8906899
C	1.5719738	0.7678409	1.8901104
C	-0.4733157	1.1308287	2.8347223
H	2.4706125	0.2729815	1.5490970
C	1.2354440	2.1211552	1.6941683
H	-1.4007171	0.9799294	3.3715055
C	-0.0317945	2.3478601	2.2745645
H	1.8493427	2.8604752	1.2003619
H	-0.5583281	3.2911577	2.3063676
C	-0.8501331	1.2990856	-2.3622586
H	-0.7143309	0.4701912	-3.0418984
C	-2.0575119	1.6664560	-1.7388983
C	0.1554658	2.1865758	-1.9224471
H	-3.0073751	1.1636135	-1.8544414
C	-1.8006178	2.7837522	-0.9157839
H	1.1952265	2.1679360	-2.2201728
C	-0.4341502	3.1093328	-1.0356065
H	-2.5290024	3.3118534	-0.3155945
H	0.0702230	3.9256596	-0.5406464
Eu	-0.4970713	0.7683457	0.2088050

7 Cartesian coordinates of model Lu–Au.

Au	-0.3436361	-1.6745571	-0.3676320
C	-2.3818796	-1.7784873	-0.3821538
C	-3.1080930	-1.7277938	-1.5786854
C	-3.1257612	-1.8727185	0.8006124
C	-4.4956697	-1.7724824	-1.5974176
H	-2.5802465	-1.6523982	-2.5250161
C	-4.5137683	-1.9161380	0.7923893
H	-2.6120348	-1.9145108	1.7563097
C	-5.2052572	-1.8652372	-0.4088756
H	-5.0254048	-1.7346224	-2.5436708
H	-5.0581452	-1.9896695	1.7282219
H	-6.2889905	-1.8979384	-0.4191234
C	1.7368803	-1.4870045	-0.2680034
C	2.4334826	-2.1852355	0.7341191
C	2.5240627	-0.6737999	-1.1011035
C	3.8028347	-2.0730412	0.8963208
H	1.8838381	-2.8338316	1.4086339
C	3.9006526	-0.5204125	-0.9199047
H	2.0715231	-0.1841502	-1.9608098
C	4.5435272	-1.2200115	0.0824419
H	4.3010955	-2.6394992	1.6759636
H	4.4639961	0.1261371	-1.5843033
H	5.6126663	-1.1168604	0.2265493
Lu	0.5605955	0.9121002	0.0474239
C	0.7783930	0.2314965	2.5103202
H	0.6551537	-0.8116368	2.7583331
C	1.9898982	0.8638096	2.1673806
C	-0.2505224	1.1971638	2.4547808
H	2.9567571	0.3856837	2.1009443
C	1.7127762	2.2232178	1.9020717
H	-1.2972347	1.0213792	2.6611829
C	0.3283808	2.4294491	2.0887253
H	2.4366673	2.9789649	1.6312173
H	-0.1937746	3.3673571	1.9748019
C	-0.1146245	1.2524259	-2.4266608
H	-0.0283868	0.4189218	-3.1088349
C	-1.2540702	1.5707141	-1.6600542
C	0.8796253	2.2177850	-2.1516728
H	-2.1764607	1.0080126	-1.6371619
C	-0.9642060	2.7255008	-0.9070847
H	1.8626794	2.2668801	-2.5994723
C	0.3572914	3.1278051	-1.2101964
H	-1.6419282	3.2253053	-0.2308154
H	0.8673180	3.9919273	-0.8100911
